# Indicators of Moderate and Severe Preeclampsia in Correlation with Maternal IL10

**DOI:** 10.3889/oamjms.2016.047

**Published:** 2016-03-23

**Authors:** Ana Daneva Markova, Marija Hadži-Lega, Dragan Mijakoski

**Affiliations:** 1 *University Clinic of Obstetrics and Gynecology, Medical Faculty, Ss Cyril and Methodius University of Skopje, Skopje, Republic of Macedonia*; 2 *Institute for Occupational Health of Republic of Macedonia – WHO Collaborating Center and GA2LEN Collaborating Center, Skopje, Republic of Macedonia*

**Keywords:** Indicators, preeclampsia, IL10, logistic regression

## Abstract

**AIM:**

The purpose of the actual study was to evaluate the relationship between the formation of anti-inflammatory cytokine IL10 and several indicators of moderate and severe preeclampsia in the third trimester of pregnancy.

**MATERIAL AND METHODS:**

Examination of the indicators of preeclampsia and maternal IL10 levels was conducted in 50 women with pregnancies complicated by varying degrees of preeclampsia in the third trimester of gestation as well as in 50 normotensive patients, hospitalized at the University Clinic of Gynecology and Obstetrics, Skopje, Republic of Macedonia. The levels of IL10 were determined with a commercial test developed by Orgenium Laboratories (Finland), using reagents from AviBion ELISA research kits. Patients with preeclampsia were categorized into moderate and severe preeclampsia group according to the degree of preeclampsia. Logistic regression analysis was used to determine the predictive value of different parameters for the occurrence of severe preeclampsia. Odds ratios and 95% Confidence Intervals were calculated in order to quantify independent associations.

**RESULTS:**

The regression analysis detected systolic blood pressure (160 mmHg or higher), diastolic blood pressure (100 mmHg or higher), persistent proteinuria in pregnancy, serum LDH concentration (450 U/L or higher) and reduced serum concentrations of IL10 as significant predictors of severe preeclampsia in pregnant women after adjusting for age.

**CONCLUSION:**

The findings of significantly lower serum IL10 concentrations in patients with severe preeclampsia in comparison with respective concentrations in patients with moderate preeclampsia can be considered as major pathognomonic laboratory sign of severe preeclampsia.

## Introduction

Accommodation of the fetoplacental unit in human pregnancy requires maternal immune tolerance to this “semiallograft”. The acceptance of the fetoplacental unit by the maternal uterine surface requires an element of immunological tolerance.

Despite close examination of the features of the process, preeclampsia remains one of the most sophisticated problems of modern obstetrics and gynecology. It generally determines maternal and perinatal morbidity and mortality [1, 2]. The role of immune mechanisms contributing to the development of normal pregnancy is widely discussed. Their involvement in the pathogenesis of pregnancy complications, such as preeclampsia, was also noted [3]. The analysis of scientific literature reveals conclusion that many aspects of the pathogenesis of preeclampsia are related to a syndrome of systemic inflammatory response characterized by development of a destructive inflammatory process, immune disorders, and imbalanced cytokine regulation of gestation processes [4-6].

The role of vascular endothelial damage with development of generalized arteriolar spasm is described as one of the leading mechanisms in the pathogenesis of preeclampsia. However, the relationship between development of endothelial dysfunction and disruption of cytokine regulation in different clinical forms of preeclampsia, currently published in several studies, also requires further research [7].

Proteinuria has been studied as both an indicator of the severity of the disease and predictor of the outcome in preeclampsia. Many clinicians still base their major management decisions on the degree of proteinuria in these patients.

In 1843, John Lever from the Guy’s Hospital in London discovered the presence of albumin by boiling the urine in pregnant women with puerperal convulsions. Preeclampsia is differentiated from gestational hypertension by the presence of proteinuria and it is the most common cause of nephrotic syndrome in pregnancy. The quantity of protein that is excreted in the urine varies widely. Significant protein excretion is defined as ≥300 mg protein in a 24-h urine collection or mark of 1+ or greater on urine dipstick testing of two random urine samples that are collected at least 4 h apart [8].

The serum uric acid level was used as an indicator of preeclampsia but it has been found that it lacks sensitivity and specificity as a diagnostic tool. However, an elevated serum uric acid level may be used in the identification of pregnant women with chronic hypertension who have an increased likelihood to develop superimposed preeclampsia [9].

A lactate dehydrogenase (LD or LDH) test is a non-specific test that may be used in the evaluation of different diseases and conditions. Thus, the blood level of LD is a general indicator of tissue and cellular damage and it is used as one of the indicators of preeclampsia [10]. Several studies have confirmed the accentuation of platelet activation in preeclampsia which remains an important obstetric complication affecting 2 to 4% of all pregnancies. Detection of aberrations of platelet function and activation appear to have predictive value for the diagnosis [11].

Studies showed that in pregnancy complicated by preeclampsia, cytokine levels essentialy change compared to the respective levels in physiological pregnancy. Thus, even a moderate form of preeclampsia shows directional change, i.e., elevated levels of pro- and anti-inflammatory cytokines, with the exception of IL-10, while a downward trend is recorded in severe preeclampsia.

The purpose of the actual study was to evaluate the relationship between formation of anti-inflammatory IL10 cytokine and several indicators of moderate and severe preeclampsia in the third trimester of pregnancy.

## Material and Methods

Examination of the indicators of preeclampsia and maternal IL10 levels was conducted in 50 women with pregnancies complicated by different degrees of preeclampsia in the third trimester of gestation as well as in 50 normotensive patients, all hospitalized at the University Clinic of Gynecology and Obstetrics, Skopje, Republic of Macedonia. Patients with preeclampsia were categorized into moderate (m PE) and severe (s PE) preeclampsia group according to the degree of preeclampsia. The severity of preeclampsia was determined according to the definition of World Health Organization, Handbook for guideline development from 2010.

Preeclampsia was defined by an increased blood pressure (≥ 140 mmHg systolic or ≥ 90 mmHg diastolic blood pressure on ≥ 2 occasions at least 6 hours apart) that occurred after the 20th week of gestation in a woman with previously normal blood pressure, accompanied by proteinuria (≥ 0.3 g/24h in the absence of urinary tract infection). Preeclampsia was defined as severe if any of the following criteria was detected: ≥ 160 mmHg systolic or ≥ 110 mmHg diastolic blood pressures or proteinuria ≥ 5.0 g/24h.

Exclusion criteria for each analyzed group included the presence of eclampsia and endocrine diseases, such as diabetes mellitus, hyper- or hypothyroidism, as well as other endocrine disorders requiring hormonal treatment. Additionally, exclusion criteria also encompassed the presence of TORCH (Toxoplasma, Rubella, Cytomegalovirus, Herpes simplex) infections markers and other chronic viral or bacterial processes in the acute phase, as well as autoimmune diseases and malignant neoplastic processes.

Patients with preeclampsia were categorized into moderate (group A) and severe (group B) preeclampsia group according to the degree of preeclampsia. Control group (group C) consisted of 50 women in the third trimester of normotensive pregnancy.

The levels of IL10 were determined with a commercial test developed by Orgenium Laboratories (Finland), using reagents from AviBion ELISA research kits. Cytokine levels in the serum were measured by a “sandwich” method of solid-phase enzyme immunoassay using double antibody. Recombinant cytokines that are part of the test whale were used as a standard for comparison of each reaction. The detection was performed by “Victor” immunoassay analytics. According to the titration of standard samples, calibration graphs were made for each cytokine, determined by their level in the range of detected concentrations (1-2000 pg/ml).

Statistical data analysis was performed using the SPSS statistical package for Windows, version 13.0. Logistic regression analysis (Binary Logistic Regression) was used to determine the predictive value of different parameters for the occurrence of severe preeclampsia. Rates of probability - odds ratios (OR) and 95% Confidence Intervals (CI) were calculated in order to quantify independent associations.

## Results

Table 1 shows significant difference in average age between pregnant women with moderate (mPE) and severe (sPE) preeclampsia (mPE 29.9 ± 4.7 years *vs*. sPE 34.2 ± 3.85 years, *p* = 0.004). Pregnant women of the analyzed groups did not differ significantly in terms of average length of gestational age, which ranges from 34.4 ± 3.6 weeks in the group with severe PE to 35.5 ± 3.4 weeks in the group with symptoms of moderate PE.

**Table 1:** Age, gestational week, BMI and IL10 serum concentration in women with moderate and severe preeclampsia, and women with normal blood pressure (control group)

Variable	Groups			
	All PE N = 50	moderate PE (mPE) N = 25	severe PE (sPE) N = 25	Control (C) N = 50
*Age* (years) mean ± SD	32.06 ± 4.8	29.9 ± 4.7	34.2 ± 3.85	31.8 ± 4.8
All PE/C; *t* = 0.27; *p* = 0.8				
mPE/sPE/C; *F* = 5.5; *p* = 0.005; post hoc mPE/sPE *p* = 0.004				
*Gestational week* mean ± SD	34.99 ± 3.5	35.5 ± 3.4	34.4 ± 3.6	34.8 ± 3.6
All PE/C; *t* = 0.2; *p* = 0.8				
mPE/sPE/C; *F* = 0.6; *p* = 0.5				
*Ethnicity n* (%) Macedonian	18 (36%)	10 (40%)	8 (32%)	15 (30%)
Albanian	28 (56%)	11 (44%)	17 (68%)	34 (68%)
Romani	4 (8%)	4 (16%)	0	1 (2%)
*BMI* mean ± SD	34.33 ± 4.5	33.1 ± 4.7	35.57 ± 4.1	32.88 ± 3.8
rang	24.2 - 44	24.2 - 41	27 - 44	27 - 43.9
All PE/C; *t* =1.7; *p* = 0.09				
mPE/sPE/C; *F* = 3.8; *p* = 0.026; post hoc sPE/C *p* = 0.025				
*IL-10* mean ±SD	23.2 ± 40.7	45.5 ± 48.4	0.8 ± 0.4	4.2 ± 6.7
median	1.36	27.28	0.75	1.47
rang	0.2 - 164	0.56 - 164	0.2 - 2.12	0.44 - 26.28
All PE/C; *Z* = 0.6; *p* = 0.5				
M PE/sPE/C; *H* = 37.68; *p* < 0.01				
*Platelets* (x10^9^/L) mean±SD	242.8 ± 68.2	268.9 ± 60.4	216.6 ± 67.4	285.2 ± 62.5
rang	100 - 401	189 - 401	100 - 320	179 - 450
mPE/C; *t* = 3.2; *p* < 0.01				
mPE/sPE/C; *F* = 9.9; *p* < 0.01				

Regarding ethnicity, Albanians represented more than a half in group with preeclampsia (44% of participants with moderate and 68% of participants with severe PE).

The average BMI in the group of pregnant women with preeclampsia was 34.33 ± 4.5 that was not significantly higher than the average BMI of the control group (32.8 8 ± 3.8) (*p* = 0.09). However, the difference between the average BMI of pregnant women with moderate and severe PE, and normotensive pregnant patients was significant (*F* = 3.8, *p* = 0.026). Namely, pregnant women with severe PE had significantly higher average BMI than normotensive pregnant women (35.57 ± 4.1 *vs*. 32.88 ± 3.8; *p* = 0.025).

Statistical analysis showed not significant differences in serum levels of IL10 between pregnant women with preeclampsia and healthy pregnant women (*p* = 0.5). The difference of IL10 serum levels between moderate preeclampsia, severe preeclampsia, and control group was highly significant (*p* < 0.01) due to:

- Lower levels of this interleukin in severe preeclampsia group,
- Differences between moderate preeclampsia and control group, and
- Significantly lower values when comparing control group in relation to moderate preeclampsia group.

Average serum concentrations of IL10 were 23.2 ± 40.7 pg/ml in the total group of preeclampsia patients, 45.5 ± 48.4 pg/ml in the group with moderate preeclampsia, and 0.8 ± 0.4 pg/ml in the group with severe preeclampsia. In patients with normal tension, the average serum concentration of IL10 was 4.2 ± 6.7 pg/ml.

Study data demonstrated that in pregnant women with pregnancy complicated by preeclampsia, the serum concentration of anti-inflammatory IL10 is confirmed as a significant predictor of the occurrence of severe preeclampsia. Increased serum concentrations of IL10 (in pg/mL) reduced the likelihood of the development of severe preeclampsia by 89.6% (95% CI 0.016-0.678).

Table 2 shows predictors of severe preeclampsia using multivariate logistic regression analysis.

**Table 2:** Multivariate logistic regression analysis for the factors predictors of severe preeclampsia

Variable	B	S.E.	Wald	*p*	Exp (B)	95% CI for Exp (B)	
Age (years)	0.2	0.086	5.350	0.021	1.221	1.031	1.446
Nulparity (present)	1.816	1.114	2.657	0.103	6.145	0.692	54.534
Systolic blood pressure (≥ 160 mmHg)	3.711	1.053	12.412	<0.001	40.9	5.189	322.371
Diastolic blood pressure (≥ 100 mmHg)	2.414	0.843	8.192	0.004	11.176	2.140	58.360
Proteinuria (present)	3.081	1.307	5.56	0.018	21.785	1.682	282.123
LDH ≥ 450 (U/L)	2.066	0.915	5.102	0.024	7.896	1.314	47.433
Albumin (serum) (g/L)	-0.239	0.125	3.66	0.056	0.787	0.616	1.006
Creatinine (serum) (umol/L)	-0.067	0.035	3.696	0.055	0.935	0.873	1.001
Platelets (≤ 150 000)	-0.006	0.013	0.236	0.627	0.994	0.97	1.019
IL10 (pg/ml)	-2.324	1.051	4.888	0.027	0.098	0.012	0.768

Dependent variable: severe preeclampsia

Table 2 presents the results of multivariate logistic regression analysis for determining the independent associations between the analyzed variables and severe preeclampsia. The variables or risk factors that showed significant predictive value within univariate analysis were included in the multivariate regression analysis. The age of the participant was used as a control variable.

The regression analysis detected systolic blood pressure (160 mmHg or higher), diastolic blood pressure (100 mmHg or higher), persistent proteinuria in pregnancy, the serum LDH concentration (450 U/L or higher) and reduced serum concentrations of IL10 as independent significant predictors of severe preeclampsia in pregnant women after adjusting for age. The age of the participant was also shown as a significant predictor of the occurrence of severe preeclampsia.

**Figure 1:**
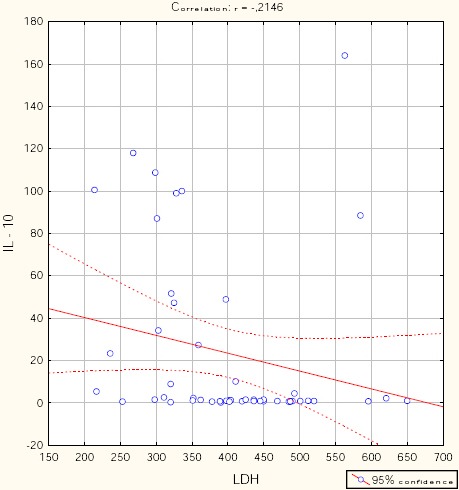
*Correlation IL10 / LDH. r = - 0.215; p = 0.134*.

**Figure 2:**
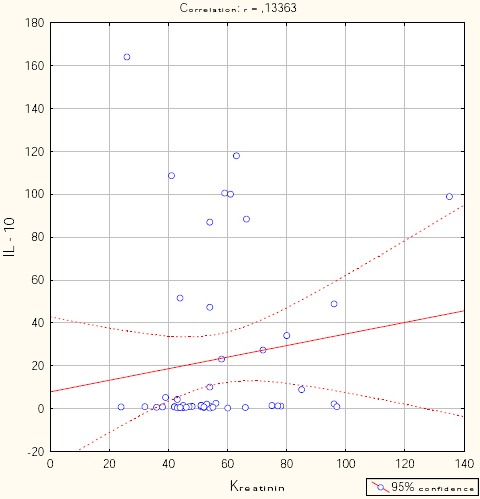
*Correlation IL10 / creatinine. r = 0.134; p = 0.355*.

**Figure 3:**
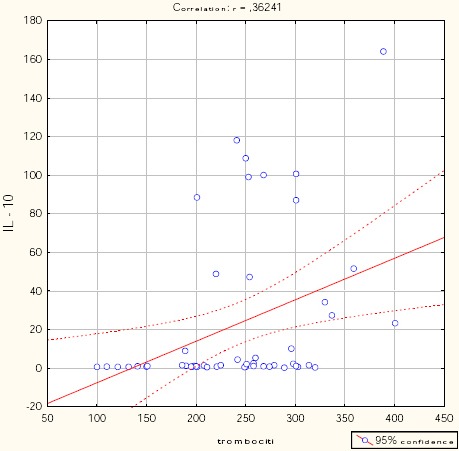
*Correlation IL10 / platelets. r = 0.362; p = 0.01*.

**Figure 4:**
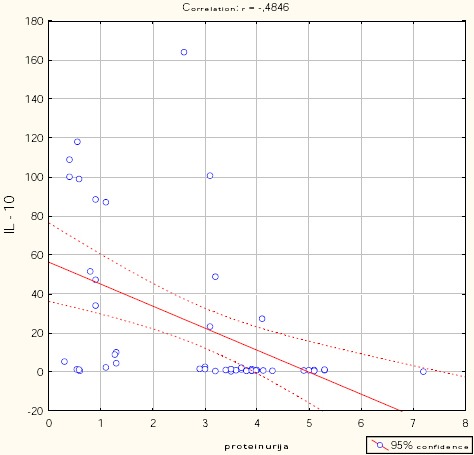
*Correlation IL10 / proteinuria. r = - 0.485; p < 0.001*.

**Figure 5:**
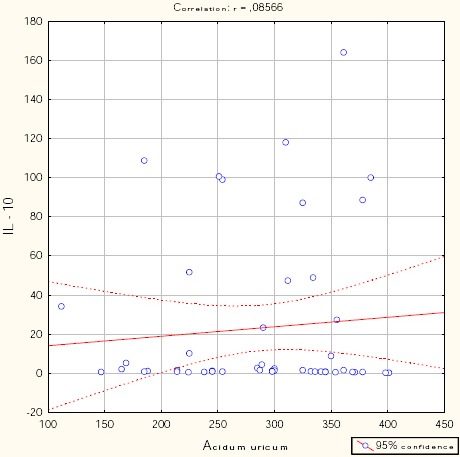
*Correlation IL10 / uric acid. r = 0.086; p = 0.554*.

Figures 1, 2, 3, 4, and 5 show the results of bivariate analysis of the relationships between serum maternal concentration of IL10 and serum enzyme LDH, creatinine, platelets, proteinuria, and uric acid, respectively.

The obtained values of Pearson’s coefficients indicate negative correlations of IL10 with LDH and proteinuria, whereas the correlations of IL10 with creatinine, platelets, and uric acid were positive. However, significant correlations were confirmed only between IL10 and platelets as well as between IL10 and proteinuria. The correlation with the platelets count was positive which means that significantly higher concentration of IL10 was confirmed in patients with higher number of platelets in the blood, and vice versa. The correlation between IL10 and proteinuria was negative showing that the serum concentration of IL10 was significantly lower in patients with higher amount of proteins in the urine, and vice versa.

Table 3 shows linear multiple regression models testing the predictive value of platelets and proteinuria for the serum concentration of IL10 in women with preeclampsia. These two parameters were confirmed in univariate analysis as variables that significantly correlate with IL10 serum concentration.

**Table 3:** Significant independent predictors of IL10 serum concentration

Adjusted *R* Square = 0.314 *R* = 0.56					*F* = 10.74 df = 2 *p*<0,001		
	Unstandardized Coefficients		Standardized Coefficients	*t*	*p*	95% CI for B	
	B	S.E.	Beta			Lower Bound	Upper Bound
Constant	11.754	21.430		0.548	0.586	-31.358	54.865
Platelets	0.169	0.073	0.285	2.325	0.024	0.023	0.316
Proteinuria	-10.099	2.857	-0.434	-3.534	0.001	-15.848	-4.351

Dependant variable: IL10 serum concentration

The results presented in Table 3 show that 31.4% of the changes of IL10 can be explained by changes in platelet count and persistent proteinuria. The platelets and proteinuria were confirmed as significant predictors that affect the serum concentration of IL10.

With each increase in the platelets count by one 10^9^ per L, the serum concentration of IL10 increases by 0.169 (95% CI for B 0.023-0.216), while any increase in urine proteins by 1 g/L, the serum concentration of IL10 is reduced by approximately 10.099 (95% CI for B -15.848; -4.351).

Beta coefficients show that greater impact on the serum concentration of IL10 has a degree of proteinuria (Beta = - 0.434).

**Figure 6:**
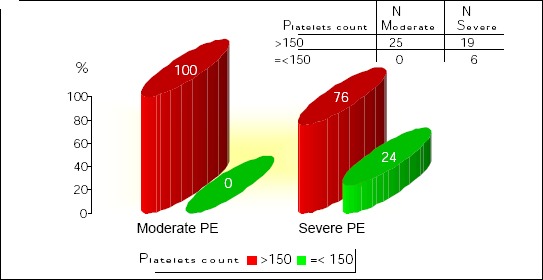
*Platelets count in moderate and severe preeclampsia. Yates Chi-square = 4.73; df = 1; p = 0.029*.

Figure 6 shows that mean platelets count is significantly lower in severe preclampsia group than in moderate preeclampsia group.

## Discussion

This study demonstrates differences in IL10 levels in women with preeclampsia compared to the levels in women with a normal pregnancy outcome.

We found that in pregnant women with preeclampsia, the increased serum concentrations of IL10 predicted lower likelihood for the development of severe preeclampsia. IL10 has been identified as an important cytokine in pregnancy. It may be involved in the maintenance of pregnancy by corpus luteum maturation and progesterone production [12]. Ovarian corpus luteum cell growth was stimulated by exogenous IL10 and also in the presence of Th2 type lymphocytes derived during early pregnancy. In a well-known mouse cross that is prone to spontaneous abortion, a deficiency of IL10 has been demonstrated to alter the net fetal number and outcome [13]. Longitudinal studies in mice demonstrate a sequential change in the cytokine profile including IL10 in peripheral blood and release from spleen elements as pregnancy advances [14, 15]. IL10 inhibition in the second half of pregnancy in mice causes fetal growth retardation [16]. Progesterone has been shown to increase Th2-type responses in T cells [17]. Taken together, these data suggest that early pregnancy is associated with an increase in circulating Th2 cytokine IL10.

This study demonstrated that there is significant alteration in the serum IL10 concentration in severe preeclampsia compared to normal pregnancy and in moderate preeclampsia group of patients.

The regression analysis applied in this study showed systolic blood pressure of 160 mmHg or higher, diastolic blood pressure of 100 mmHg or higher, persistent proteinuria in pregnancy, the serum LDH concentration of 450 U/L or higher and reduced serum concentrations of IL10 as significant predictors of severe preeclampsia in pregnant women. While other variables predicted higher likelihood for the development of severe preeclampsia, IL10 decreased such likelihood. IL10 was also found to be negatively correlated with proteinuria, and positively correlated with blood platelets. Significantly higher concentration of IL10 was confirmed in patients with higher number of platelets in the blood, and vice versa. On the other hand, serum concentration of IL10 was significantly lower in patients with higher amount of proteins in the urine, and vice versa.

The actual study demonstrated platelets count and proteinuria as significant predictors of serum IL10 concentration - platelets count predicting higher serum concentration of IL10, while urine proteins predicting lower serum IL10.

Some studies suggest a proportional link between the level of proteinuria and adverse clinical outcomes. Page et al., in a prospective study of almost 13,000 pregnant women found that significant proteinuria, defined as 2+ or more on dipstick analysis, was associated with an increase in stillbirth rates, fetal growth restriction and neonatal morbidity, when associated with hypertension [18]. Other studies suggest that it is the presence of proteinuria rather than the severity, which is associated with poorer outcomes. There is evidence that even the finding of trace proteinuria in pregnant women with hypertension is associated with an increase in adverse outcome [19].

This may have contributed to the variation in the diagnostic performance among the studies [20]. Significant proteinuria of 150-180 mg/24h urine has been established in the preeclamptic group of this study which is similar to finding of Nisell et al. (1995), Lindheir et al. (1986) and Davison (1986) who reported 300 mg/24h of protein in urine of preeclamptic women, while Waugh et al. (2003) between 150-200 mg/24h and Higby et al. (1995) who recorded between 120-200 mg/24h.

The mean platelets count was significantly lower in severe preclampsia group than in moderate preeclampsia group. This study showed decrease of platelets counts among the preeclamptic group and this is consistent with the findings of other studies [21, 22], demonstrating that platelet count was significantly lower in severe preeclampsia groups. This results proposes a possible relationship between the platelet count and the severity of preeclampsia. In our study the correlation of IL10 with the platelets count was positive.

Taking into consideration changes of anti-inflammatory cytokine concentrations in severe preeclampsia, moderate phase can be analyzed as a critical stage in complicated pregnancies.

It can be assumed that moderately aggressive factors have a role as initiators of synthesis of mediators of intercellular interaction (in moderate preeclampsia) and the development of immune response is regulated by the interaction of cytokines and their antagonists. With increasing severity of the pathological process, the impact of regulatory factors that limit the systemic effect is reduced.

In conclusion, there are dynamic changes in maternal concentrations of cytokines in normotensive and preeclampsia pregnancy. The findings of significantly lower serum IL10 concentrations in patients with severe preeclampsia in comparison with respective concentrations in patients with moderate preeclampsia are highly important. These results can be considered as a major pathognomonic laboratory sign of severe preeclampsia that can be used by clinicians to make difference between severe preeclampsia and normal pregnancy, as well as between severe and moderate degree of this specific pathology. The decrease in serum IL10 concentrations indicates a depletion of adaptive mechanisms aimed at relief of excessive activity of the inflammatory process and development of physilogical immunosuppression during pregnancy and, apparently, can be an additional diagnostic criterion for the prediction and assessment of the severity of the pathological process.

These indicators may help in recognizing patients with the highest risk of severe preeclampsia. Precisely defined time for the termination of preeclampsia pregnancy will decrease morbidity and mortality from this most difficult disease in pregnancy.
